# The effects of melatonin treatment on oxidative stress induced by ovariohysterectomy in dogs

**DOI:** 10.1186/s12917-021-02882-1

**Published:** 2021-05-01

**Authors:** Sina Salavati, Asghar Mogheiseh, Saeed Nazifi, Atefeh Amiri, Behrooz Nikahval

**Affiliations:** Department of Clinical Sciences, School of Veterinary Medicine, Shiraz University, Shiraz, Fars 7144169155 Iran

**Keywords:** Dog, Surgery, Oxidative stress, Melatonin, Antioxidants

## Abstract

**Background:**

As one of the most common surgeries performed in veterinary medicine, ovariohysterectomy (OHE) can induce oxidative stress in dogs. The antioxidant properties of melatonin have been confirmed in various studies. This study aimed to investigate the effects of melatonin administration on oxidative stress in dogs before and after OHE. In this study, 25 mature female intact dogs were selected and randomly divided into five equal groups: Melatonin (melatonin, no surgery), OHE (no melatonin, surgery), OHE + melatonin (melatonin, surgery), anesthesia+melatonin (melatonin, sham surgery), and control (no melatonin, no surgery) groups. Melatonin (0.3 mg/Kg/day, p.o.) was administrated to the dogs in the melatonin, OHE + melatonin, and anesthesia+melatonin groups on days − 1, 0, 1, 2, and 3 (day 0 = OHE). Blood sampling was performed on days − 1, 1, 3, and 5 of the study. Blood samples were immediately transferred to the laboratory and sera were separated and stored at − 20 °C. Superoxide dismutase (SOD), glutathione peroxidase (GPX), catalase (CAT) and malondialdehyde (MDA) concentrations were measured with commercial kits.

**Results:**

The levels of SOD, GPX and CAT were significantly higher in the melatonin and anesthesia+melatonin groups compared to those of the control group at days 3 and 5. The level of antioxidant enzymes significantly decreased in the OHE group compared to that of other groups at days 3 and 5. The administration of melatonin increased the level of antioxidant enzymes in ovariohysterectomized dogs. Ovariohysterectomy significantly increased the concentration of MDA in comparison to that of other groups at day 3. Melatonin administration significantly decreased the level of MDA in melatonin, anesthetized, and ovariohysterectomized dogs at day 3.

**Conclusions:**

Administration of melatonin on day − 1, 0, 1, 2 and 3 modulate the oxidative stress induced by OHE in dogs by increasing antioxidant enzymes concentration and decreasing MDA levels.

## Background

Increased reactive oxygen species (ROS) can cause damage to macromolecules (lipids and proteins), lead to cell damage, and decrease ROS scavenging mechanisms, resulting in oxidative stress [1]. There are several markers that can be used to investigate the changes in the oxidative status. These markers are substances that are formed as a result of the reaction between ROS and biomolecules. One of these substances produced through the above reaction is malondialdehyde (MDA), which is a useful indicator of lipid peroxidation [2–4]. One of enzymatic antioxidants is catalase (CAT), which catalyzes hydrogen peroxide detoxification [3, 5]. Glutathione is a non-enzymatic antioxidant that is involved in vitamin metabolism and plays a key role in cell membrane protection [2, 6]. As antioxidant enzymes, Glutathione peroxidase (GPX) and superoxide dismutase (SOD) activate the enzymatic defense system against ROS [2, 7].

In recent years, oxidative stress markers have been measured after spaying and the markers have been used to identify possible solutions to reduce oxidative stress [8]. As an elective and sometimes urgent method of spaying, ovariohysterectomy (OHE) has been reported to have some early side effects, such as increased oxidative stress due to traumatic events during surgery, ischemia, increased levels of free iron and copper released from tissues, and inflammation of serous membranes [2, 3, 9]. The balance between oxidants and antioxidants is crucial for living organisms to remain healthy and functional [10].

As a neurotransmitter, melatonin (N-acetyl-5-methoxytryptophan) is a hormone produced and released by the pineal gland or epiphysis during the night in a circadian rhythm [11]. Under special conditions, the neuroendocrine cells of other organs, including the thyroid gland, retina, skin, and gastrointestinal tract, can also produce melatonin in vertebrates [12]. In our previous experiments, we found that melatonin could have anti-oxidative, anti-inflammatory, and anti-stress (based on cortisol level) effects and regulate the secretion of metabolic hormones in dogs following castration [13–16].

Due to the antioxidant effects of melatonin and the presence of oxidative stress in dogs undergoing open ovariectomy and OHE [7, 9, 17, 18], the present study aimed to evaluate the effects of melatonin treatment on oxidative stress before and after OHE and anesthesia.

## Results

**Fig. 1:**
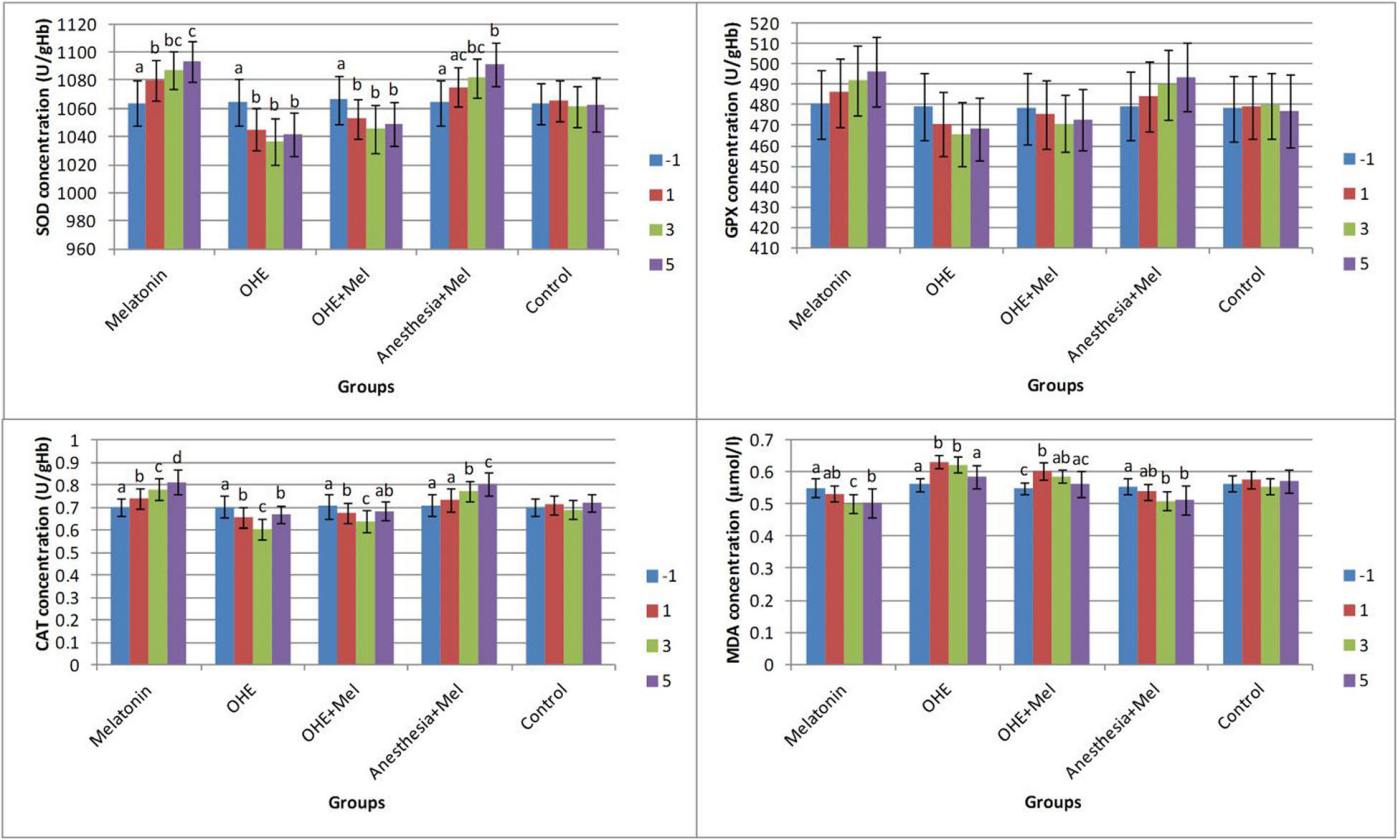
The mean ± SD concentrations of superoxide dismutase (SOD), glutathione peroxidase (GPX), catalase (CAT), and malondialdehyde (MDA) were compared between days of sampling in each group. Melatonin (0.3 mg/kg p.o.) was administrated on days -1, 0, 1, 2 and 3. Ovariohysterectomy and anesthesia were performed on day 0. Different letters on bars indicate significant differences between days of sampling in each group

**Fig. 2:**
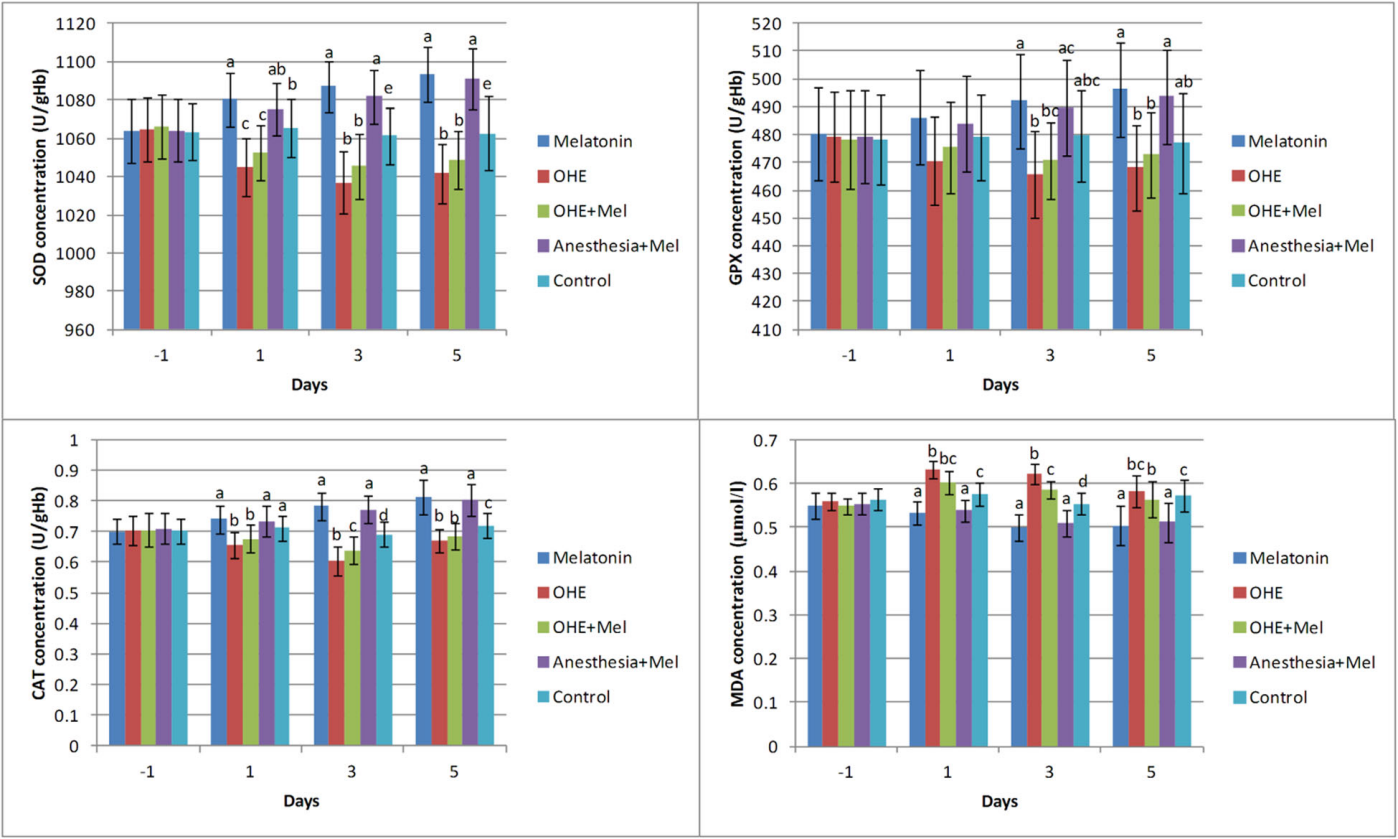
The mean ± SD concentrations of superoxide dismutase (SOD), glutathione peroxidase (GPX), catalase (CAT), and malondialdehyde (MDA) were compared between the study groups (melatonin, ovariohysterectomy (OHE), OHE+melatonin, anesthesia+melatonin, and control) in each day of sampling. Melatonin (0.3 mg/kg p.o.) was administrated on days -1, 0, 1, 2 and 3. Ovariohysterectomy and anesthesia were performed on day 0. Different letters (**a**, **b**, **c**, **d**) on bars indicate significant differences (*P* < 0.05) between groups on each day of sampling

There was no difference in age, body weight, duration of surgery, peri- or post-operative complications between groups. The effects of time (days of sampling; Fig. 1) and group (melatonin, OHE, OHE + melatonin, anesthesia+melatonin, and control; Fig. 2) were analyzed for each factor in this study.

### SOD level

There was no statistically significant difference in SOD concentrations between groups on day − 1. SOD concentrations significantly decreased after OHE (day − 1 vs. days 1, 3, and 5, *P* < 0.01) in the OHE and OHE + melatonin groups. Melatonin administration significantly increased the SOD concentration in melatonin (day − 1 vs. days 1, 3, and 5 (*P* < 0.002) and day 1 vs. day 5 (*P* = 0.02)) and anesthesia+melatonin groups (day − 1 vs. days 3 and 5, (*P* < 0.003)) (Fig. 1).

The SOD concentration decreased significantly in the ovariohysterectomized dogs on days 1, 3 and 5 after surgery in comparison with melatonin treated and control groups (*P* < 0.03). There were no significant changes in SOD concentration between melatonin vs. anesthesia+melatonin, OHE vs. OHE + melatonin (day 1, 3 and 5), and anesthesia+melatonin vs. control (day 1) (Fig. 2).

### GPX level

On days − 1 and 1 of sampling, there was no significant difference in GPX concentration between groups. Interestingly, no significant difference in GPX concentration was observed between days of sampling (− 1, 1, 3 and 5) in all groups (Fig. 1).

The GPX concentration was significantly decreased in OHE group in comparison with melatonin and anesthesia+melatonin groups (day 3, 5, (*P* < 0.01)). The GPX concentration significantly increased in melatonin and anesthesia+melatonin groups vs. OHE and control groups (day 3, 5, *P* < 0.04) (Fig. 2).

### CAT level

On day − 1, the CAT concentration was not significantly different between groups. The CAT concentration significantly increased in the melatonin (between day − 1, 1, 3, 5, *P* < 0.01) and anesthesia+melatonin groups (between day − 1 and 1 vs. 3 and 5, *P* < 0.01). The CAT concentration significantly decreased in OHE (between day − 1, 1, 3, *P* < 0.01) and OHE + melatonin groups (between day − 1, 1, 3, *P* < 0.03) (Fig. 1).

On days 1, 3 and 5, OHE significantly decreased the CAT concentration in the OHE and OHE + melatonin groups in compared with other groups (*P* < 0.03). Also, the CAT concentration significantly increased in the melatonin and anesthesia+melatonin in compared with other groups on days 3 and 5 (*P* < 0.0001) (Fig. 2).

### MDA level

On day − 1 of sampling, the MDA concentration was not significantly different between groups. The concentration of MDA was significantly decreased in the melatonin between day − 1 vs. 3 and 5 and between day 1 vs. day 3 (*P* < 0.02) and anesthesia+melatonin groups between day − 1 vs. days 3 and 5 (*P* < 0.002). A significant increase in the concentration of MDA was observed in OHE and OHE + melatonin groups between day − 1 vs. 1 and 3 (*P* < 0.006; Fig. 1).

The MDA concentration was significantly decreased in melatonin and anesthesia+melatonin groups vs. OHE, OHE + melatonin, and control groups (days 1, 3 and 5, *P* < 0.01). The MDA concentration was significantly higher in OHE and OHE + melatonin in compared with melatonin and anesthesia+melatonin (days 1, 3 and 5, *P* < 0.04; Fig. 2).

## Discussion

A significant decrease in the serum concentration of antioxidant enzymes (SOD, GPX and CAT) along with a significant increase in the serum concentration of MDA were found in dogs after OHE. According to the present results, melatonin treatment could increase the expression of antioxidant enzymes (SOD, GPX, and CAT) and significantly decrease the MDA level after OHE. Melatonin partially reduced the oxidative stress in dogs with OHE, as suggested by a decrease in the concentration of SOD, GPX, and CAT in all the groups that received melatonin. The activities of antioxidant enzymes significantly increased in the anesthesia group that received melatonin. In addition, in the melatonin-treated groups, melatonin led to a decrease in the level of MDA, which had reached its peak (MDA concentration) 3 days after surgery.

Other studies showed increased lipid peroxidation 24 h after surgery [2, 19]. In another study, the MDA level reached its peak 3 days after OHE in dogs but the CAT level did not differ significantly between experimental days. Also, the researchers indicated that the oxidative stress caused by OHE and plasma MDA was higher on day 3 post-OHE than before OHE and 3-h and 3–14 days post-OHE. Total antioxidant capacity on day 14 post-OHE was higher than pre-OHE, 3 h and 3–14 days post OHE. They also suggested the ovariohysterectomized dogs needed antioxidant for 7 days post-OHE to protect their bodies from free radicals [3].

Furthermore, researchers were observed that OHE and anesthesia, induced by a combination of Ketamine-Xylazine, could lead to an increased in the oxidative stress markers (Glutathione and MDA). The toxicity of drugs commonly used in anesthesia can induce oxidative stress. A significant decrease in antioxidative capacity was reported in dogs after enflurane anesthesia. It was observed that the serum level of vitamin E and beta-carotene significantly decreased whereas the serum level of vitamin A and MDA significantly increased during enflurane anesthesia in dogs [20].

Several studies have reported an oxidative stress induced by OHE in dogs and efforts should be made to identify potential antioxidant therapies [3, 18]. Melatonin has been shown in numerous studies to have antioxidant properties in dogs and human [14, 21]. In dogs, oral melatonin (0.3 mg/kg) was administrated daily for 1 month after castration [14]. Their findings indicated that antioxidant enzymes (GPX, CAT and SOD) increased and MDA concentration decreased in dogs treated with melatonin in compared with control and castrated dogs [14]. Melatonin increased antioxidant enzymes activity in different tissues (brain, liver, and kidney) during physiological (circadian rhythm) and pathological conditions. Furthermore, melatonin has been shown to regulate the activation or inhibition of several transcription factors related to antioxidant response, which can justify the antioxidative effects of this substance [21]. According to a study, male dogs were not under oxidative stress after castration due to the non-significant changes in the MDA and CAT concentrations. They were observed a decrease in total antioxidant capacity (TAC) on days 3, 7, and 10 compared to day 14, but their antioxidant system still functioned with high effectiveness [22]. OHE under ketamine-xylazine anesthesia was found to increase lipid peroxidation and decreased antioxidant enzyme activity in rats [23].

Other strategies have been previously studied in an attempt to identify potential treatments to reduce the oxidative stress induced by surgery or anesthesia. The researchers investigated the physiologic effects of hyperbaric oxygen (HBO) therapy on the antioxidant status in healthy dogs after OHE. They found that while this therapy, at the dose used in their study, had no adverse effects on the treated group, it was ineffective in reducing postoperative inflammation and improving the oxidative status in them [17]. In the study on the effects of flunixin meglumine (FM) and meloxicam on postoperative and oxidative stress after OHE, it is reported that while FM reduced postoperative oxidative stress, it had no influence on the oxidative stress status [24]. Elevated fraction of inspired oxygen during and after general anesthesia significantly increased MDA concentration 24 h after surgery and decreased the body antioxidant defense marker and glutathionyl hemoglobin in human [25].

In our previous study, we examined the effects of melatonin treatment on the levels of sexual hormones, serotonin, and cortisol in intact and castrated male dogs. The results showed that melatonin increased the level of serotonin but decreased the level of cortisol in intact and castrated dogs [13]. This study indicated that postoperative stress (increased cortisol concentration) continued for 1 week after castration and daily melatonin administration decreased cortisol concentration during 7 days after gonadectomy in castrated dogs. Thus, the authors suggested that melatonin administration considered pre- and post-operation period for about 7 days after OHE in the bitches. We also studied the effects of melatonin administration on the levels of thyroid hormones, leptin, and ghrelin in intact and castrated male dogs. Melatonin could regulate metabolic hormones and mitigate the metabolic side-effects of castration [15]. Long-term side effects of castration and beneficial effect of melatonin were reported in these studies [13, 15]. The melatonin treatment after gonadectomy may be suggested for prevention of metabolic disorders in gonadectomized dogs.

Malondialdehyde (MDA) represents one of the most investigated end products of lipid oxidation. Despite thiobarbituric acid test for MDA determination being the most frequently used method to evaluate lipid peroxidation, it has several pitfalls including thiobarbituric acid reaction with several compounds, including sugars, amino acids, bilirubin, and albumin, and the interference of hemolysis that falsely increases the measured MDA levels [26]. The most important antioxidant enzymes are SOD, CAT, and glutathione-dependent enzymes, such as GPX, and glutathione transferases. Changes of antioxidant enzymes activity are dependent on age, obesity, disease and stage of disease, different samples, processing and cryopreservation procedures and different cell types of blood [26]. Various methods were used for measuring nonenzymatic antioxidants (nonenzymatic antioxidant capacity, also named total antioxidant capacity) and included endogenous (e.g., urine analysis, bilirubin, and thiols) and nutritional (e.g., tocopherols, ascorbic acid, carotenoids, and phenolics) compounds in plasma. The measuring of the antioxidant enzymes activity can be replaced with total antioxidant status assay [26].

In this study, melatonin administration decreased oxidative stress in the ovariohysterectomized bitches. It may reduce the side effects of the surgery. It is suggested to measure melatonin concentration in dogs. Also, melatonin treatment included in more aggressive surgeries than castration or OHE, such as orthopedics and evaluated its effects in much more oxidative stress and stressful conditions. Bioavailability of oral melatonin following a 10 mg/kg dose is moderate in rats and high in dogs and monkeys. However, the bioavailability appears to be dose dependent, at least in dogs and monkeys [27]. Therefore, further studies are needed to determine the most appropriate therapeutic dose of oral melatonin in dogs to achieve an antioxidant effect.

The small sample size (*n* = 5) in this study was considered because of minimal number of repeats in each group to provide one of the essential condition for performing parametric statistical tests and regarding the standards in the protection of animals [28]. Increasing sample size will be helpful to decrease type II error. Injection of NSAIDs following surgery may influence oxidative stress by pain relief effect (pain can induce oxidative stress) [29]. Therefore, in the next studies, it needs to be considered and compared the effect of NSAIDs on oxidative stress with or without melatonin treatment in experimental design.

## Conclusion

In summary, melatonin administration at a daily oral dose of 0.3 mg/kg 1 day before and for 3 days after OHE surgery increased the activity of antioxidant enzymes and decreased the level of MDA, a lipid peroxidation index, leading to reduced oxidative stress. Melatonin was also found to improve the antioxidant status of dogs after anesthesia. Further studies are needed to determine the potential role of melatonin and an optimal protocol of administration to regulate oxidative stress in dogs with various diseases.

## Methods

This experiment was performed in accordance with the Iranian animal ethics framework under the supervision of the Iranian Society for the Prevention of Cruelty to Animals and Shiraz University Research Council (IACUC no: 4687/63).

### Animals

Twenty-five clinically healthy mixed-breed adult female dogs, between 1 and 3 year-old and weighing 15–20 kg, were used in this study. The dogs were owned and taken care of by Shiraz University School of Veterinary Medicine. All the dogs were ovariohysterectomized at the end of the study and kept in a non-governmental organization shelter for adoption. All dogs were fed with commercial dog food (300 g/dog/day; Nutripet™; Behintash Co., Karaj, Iran) and given ad libitum access to water. The dogs were kept under a controlled condition with 12:12 h light/dark cycles. During the first 2 weeks of acclimatization, the animals were treated with antiparasitics (fenbendazole, 150 mg; pyrantel embonate, 144 mg; praziquantel, 50 mg; Caniverm**®**, 0.7 mg/10 kg, p.o.). The overall health of dogs was evaluated daily during the study by checking their body temperature, heart rate, respiratory rate, appetite, and behavior at the time of feeding and cleaning of their shelter. The pregnancy status of dogs was examined by transabdominal ultrasonography.

### Experimental design

**Fig. 3:**
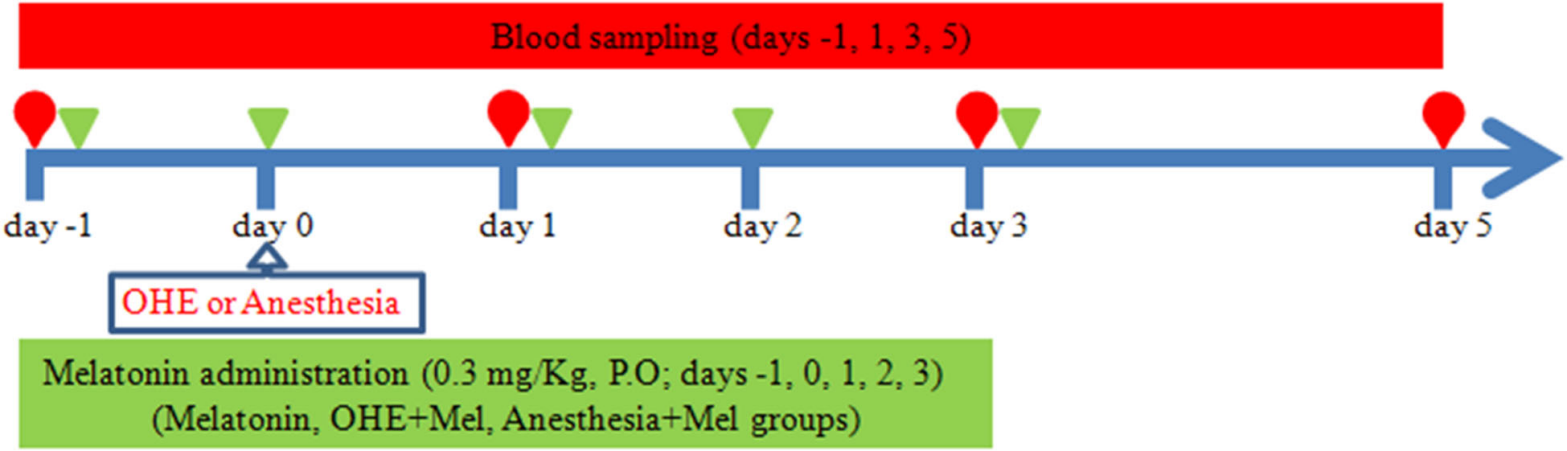
The schematic design of the study. Blood sampling on days -1, 1, 3, and 5 was performed before oral melatonin administration (0.3 mg/kg) on days -1, 0, 1, 3, and 5 of the study. Ovariohysterectomy (OHE) and anesthesia were performed on day 0

The Oral melatonin (3 mg, L’ORGANIQUE, Nutralab Canada LTD, Canada) was administrated daily, the day before (day − 1), the day of OHE (day 0) and 1, 2 and 3 days after OHE (day 1, day 2 and day 3, respectively), with a meal. Also, to examine the effect of OHE, melatonin and anesthesia on oxidative stress [13–16], the dogs were divided into five equal groups (*n* = 5) in a case-control randomized study (Fig. 3). The dogs in the OHE-melatonin group were spayed on day 0, and received melatonin on day − 1, day 0, day 1, day 2 and day 3. The melatonin group (melatonin, no surgery) received melatonin (0.3 mg/kg, p.o.) once daily on days − 1, 0, 1, 2, and 3. The dogs in the OHE group (no melatonin, surgery) were spayed on day 0 but did not receive melatonin. The dogs in the anesthesia+melatonin group (melatonin) were anesthetized on day 0 and received melatonin on days − 1, 0, 1, 2, and 3. The control group (no melatonin, no surgery) was neither spayed nor received melatonin during the study. Blood sampling was collected from the jugular vein into simple glass tubes and ethylenediaminetetraacetic acid (EDTA) vacutainer tubes on days − 1, 1, 3, and 5 (Fig. 3). The serum was separated from each blood sample by centrifugation at 750×g for 10 min and stored at − 20 °C until laboratory assays. Also, for assaying the glutathione peroxidase (GPX), superoxide dismutase (SOD), and catalase (CAT) activity, red blood cell (RBC) washing was performed three times by adding 3 ml of normal saline to 0.5 ml of blood samples from EDTA vacutainers and centrifuged for 10 min and 3000 × rpm. After the last washing and removal of the supernatant, 2 ml of distilled water were added to the RBC. The samples were kept at 4 °C for 15 min and stored at − 20 °C.

### Ovariohysterectomy

Ten dogs were ovariohysterectomized under general anesthesia on day 0. The animals were kept off food and water for 12 h prior to the surgery. The monitoring used during surgery was at the discretion of the anesthesiologist. The dogs were premedicated with acepromazine (0.05 mg/kg, i.m.) and xylazine (0.5 mg/kg, i.m.). Anesthesia was induced with ketamine (5 mg/kg, i.v.) and diazepam (0.25 mg/kg, i.v.). After endotracheal intubation, general anesthesia was maintained with isoflurane (1.2%) vaporized in oxygen using intermittent positive pressure ventilation. Ketoprofen (1 mg/kg, i.m.) and ampicillin (20–10 mg/kg, i.m.) were administered at the induction and the end of surgery [30].

### Laboratory measurements

The following commercial kits were used to colorimetrically measure the activity levels of SOD, GPX, and CAT (ZellBio Company, Germany; SOD, Cat. No. ZB-SOD-96A; GPX, Cat. No. ZB-GPX-96A; CAT, Cat. No. ZB-CAT-96A) at 420, 412, and 540 nm, respectively in hemolyzed RBCs. The assay kit sensitivity was 1 unit/ml (inter-assay coefficients of variability (CV): 5.8%) for SOD, 5 unit/ml (inter-assay CV: 3.5%) for GPX and 0.5 unit/ml (inter-assay CV: 6.3%) for CAT. The activities of the antioxidant enzymes were all stated as units per gram of hemoglobin. All kits were validated for dog samples in the laboratory.

An assay kit purchased from ZellBio GmbH (Germany) was used to measure MDA (μmol/L; Cat. no. ZB-MDA-96A). In this kit, MDA is measured based on its reaction with thiobarbituric acid in an acidic condition and high temperature. The color complex was measured colorimetrically at 535 nm. The assay kit sensitivity was 0.1 μM (inter-assay CV: 5.8%) for MDA.

### Statistical analysis

All data are presented as mean ± standard deviation (SD). Normal distribution was confirmed by analyzing the data with Kolmogorov–Smirnov test. The statistical analysis of the data was performed by two-way repeated-measures ANOVA and Tukey’s test using GraphPad Prism version 6 (GraphPad Software, San Diego, California, USA). The significant level was set at *P* < 0.05.

## Data Availability

The datasets generated and/or analysed during the current study are available from the corresponding author on request.

## Abbreviations

OHE: Ovariohysterectomy
SOD: Superoxide dismutase
GPX: Glutathione peroxidase
CAT: Catalase
MDA: Malondialdehyde
ROS: Reactive oxygen species
RBC: Red blood cell

## References

[1] Gutteridge JM (1995). Lipid peroxidation and antioxidants as biomarkers of tissue damage. Clin Chem.

[2] Serin G, Kiral F, Serin I (2008). Acute effect of ovariohysterectomy on lipid peroxidation and some antioxidant levels in dogs. Bull Vet Inst Pulawy.

[3] Sakundech K, Chompoosan C, Tuchpramuk P, Boonsorn T, Aengwanich W (2020). The influence of duration on pain stress, oxidative stress, and total antioxidant power status in female dogs undergoing ovariohysterectomy. Vet World.

[4] Khoubnasabjafari M, Ansarin K, Jouyban A (2015). Reliability of malondialdehyde as a biomarker of oxidative stress in psychological disorders. BioImpacts..

[5] Mehaney DA, Darwish HA, Hegazy RA, Nooh MM, Tawdy AM, Gawdat HI (2014). Analysis of oxidative stress status, catalase and catechol-O-methyltransferase polymorphisms in Egyptian vitiligo patients. PLoS One.

[6] Hayden RE, Paniello RC, Yeung CS, Bello SL, Dawson SM. The effect of glutathione and vitamins A, C, and E on acute skin flap survival. Laryngoscope. 1987;97(10):1176–9. 10.1288/00005537-198710000-00011 3657365

[7] Gogoi J, Leela V, Suganya G, Shafiuzama M, Vairamuthu S, Rajamanickam K (2018). Effect of ovariohysterectomy on oxidative stress markers in pyometra affected bitches. Int J Chem Stud.

[8] Michelsen J, Heller J, Wills F, Noble G (2012). Effect of surgeon experience on postoperative plasma cortisol and C-reactive protein concentrations after ovariohysterectomy in the dog: a randomised trial. Aust Vet J.

[9] Lee JY, Kim MC (2014). Comparison of oxidative stress status in dogs undergoing laparoscopic and open Ovariectomy. J Vet Med Sci.

[10] Tomsič K, Seliškar A, Lukanc B, Svete AN (2016). Plasma total antioxidant capacity and activities of blood glutathione peroxidase and superoxide dismutase determined in healthy dogs by using commercially available kits. Acta Vet.

[11] Yoshikawa T, Iigo M, Okano T, Fukada Y. Reproductive Biology and Clinical Endocrinology; Roles of Melatonin in Photoperiodic Gonadal. In: Haldar C, Singaravel M, Pandi-Perumal S, editors. Experimental endocrinology and reproductive biology. 1st ed. Taylor & Francis; 2008.

[12] Ambriz-Tututi M, Rocha-González HI, Cruz SL, Granados-Soto V (2009). Melatonin: a hormone that modulates pain. Life Sci.

[13] Salavati S, Mogheiseh A, Nazifi S, Tabrizi AS, Taheri P, Koohi F (2018). Changes in sexual hormones, serotonin, and cortisol concentrations following oral administration of melatonin in castrated and intact dogs. J Vet Behav.

[14] Mogheiseh A, Koohi F, Nazifi S, Tabrizi AS, Taheri P, Salavati S (2019). Oxidative-antioxidative status and hepatic and renal factors following melatonin administration in castrated and intact dogs. Basic Clin Androl.

[15] Taheri P, Mogheiseh A, Tabrizi AS, Nazifi S, Salavati S, Koohi F (2019). Changes in thyroid hormones, leptin, ghrelin and, galanin following oral melatonin administration in intact and castrated dogs: a preliminary study. BMC Vet Res.

[16] Nazifi S, Mogheiseh A, Tabrizi AS, Rayat MH (2020). Effect of oral melatonin administration on inflammatory cytokines and acute phase proteins after the castration of dogs. Comp Clin Pathol.

[17] Gautier A, Graff EC, Bacek L, Fish EJ, White A, Palmer L (2020). Effects of ovariohysterectomy and hyperbaric oxygen therapy on systemic inflammation and oxidation in dogs. Front Vet Sci.

[18] Gunay A, Gunes N, Gunay U (2011). Effect of ovariohysterectomy on lipid peroxidation and levels of some antioxidants and biochemical parameters in bitches. Bull Vet Inst Pulawy.

[19] Sane A, Chokshi S, Mishra V, Barad D, Shah V, Nagpal S (1993). Serum lipoperoxide levels in surgical stress of abdominal hysterectomy. Panminerva Med.

[20] Naziroglu M, Günay C (1999). The levels of some antioxidant vitamins, glutathione peroxidase and lipoperoxidase during the anaesthesia of dogs. Cell Biochem Funct.

[21] Rodriguez C, Mayo JC, Sainz RM, Antolín I, Herrera F, Martín V (2004). Regulation of antioxidant enzymes: a significant role for melatonin. J Pineal Res.

[22] Aengwanich W, Sakundech K, Chompoosan C, Tuchpramuk P, Boonsorn T (2019). Physiological changes, pain stress, oxidative stress, and total antioxidant capacity before, during, and after castration in male dogs. J Vet Behav.

[23] Elvan A (2016). Effect of ovariohysterectomy on some oxidative stress markers in the rat. Harran Univ Vet Fak Derg.

[24] Yilmaz O, Korkmaz M, Jaroszewski J, Yazici E, Ulutas E, Saritas Z (2014). Comparison of flunixin meglumine and meloxicam influence on postoperative and oxidative stress in ovariohysterectomized bitches. Pol J Vet Sci.

[25] Ottolenghi S, Rubino FM, Sabbatini G, Coppola S, Veronese A, Chiumello D (2019). Oxidative stress markers to investigate the effects of Hyperoxia in anesthesia. Int J Mol Sci.

[26] Marrocco I, Altieri F, Peluso I (2017). Measurement and clinical significance of biomarkers of oxidative stress in humans. Oxidative Med Cell Longev.

[27] Yeleswaram K, McLaughlin LG, Knipe JO, Schabdach D (1997). Pharmacokinetics and oral bioavailability of exogenous melatonin in preclinical animal models and clinical implications. J Pineal Res.

[28] Brown MJ, Winnicker C, Fox JG, Anderson LC, Otto GM, Pritchett-Corning KR, Whary MT (2015). Chapter 39 - animal welfare. Laboratory animal medicine.

[29] Tavakoli A, Tazik ME, Abbasi A. Comparison of production of pain and oxidative stress after induction of local nerve block or use of NSAIDs following painful dental procedures in dogs. Iranian J Vet Surg. 2021;16(1):29–33.

[30] Fossum TW, Fossum TW, Duprey LP, Huff TG (2018). Surgery of the reproductive and genital systems. Small animal surgery.

